# BDNF, NT-3 and Trk receptor agonist monoclonal antibodies promote neuron survival, neurite extension, and synapse restoration in rat cochlea ex vivo models relevant for hidden hearing loss

**DOI:** 10.1371/journal.pone.0224022

**Published:** 2019-10-31

**Authors:** Stephanie Szobota, Pranav D. Mathur, Sairey Siegel, KristenAnn Black, H. Uri Saragovi, Alan C. Foster

**Affiliations:** 1 Otonomy, Inc., San Diego, California, United States of America; 2 Lady Davis Institute-Jewish General Hospital, McGill University, Montreal, Quebec, Canada; University of Louisville, UNITED STATES

## Abstract

Neurotrophins and their mimetics are potential treatments for hearing disorders because of their trophic effects on spiral ganglion neurons (SGNs) whose connections to hair cells may be compromised in many forms of hearing loss. Studies in noise or ototoxin-exposed animals have shown that local delivery of NT-3 or BDNF has beneficial effects on SGNs and hearing. We evaluated several TrkB or TrkC monoclonal antibody agonists and small molecules, along with BDNF and NT-3, in rat cochlea ex vivo models. The TrkB agonists BDNF and a monoclonal antibody, M3, had the greatest effects on SGN survival, neurite outgrowth and branching. In organotypic cochlear explants, BDNF and M3 enhanced synapse formation between SGNs and inner hair cells and restored these connections after excitotoxin-induced synaptopathy. Loss of these synapses has recently been implicated in hidden hearing loss, a condition characterized by difficulty hearing speech in the presence of background noise. The unique profile of M3 revealed here warrants further investigation, and the broad activity profile of BDNF observed underpins its continued development as a hearing loss therapeutic.

## Introduction

Spiral ganglion neurons convey sensory information from the hair cells of the cochlea to the brain stem and are vulnerable to damage from noise, aging, and underlying genetic diseases. In animal models of noise trauma, recent studies have shown that SGN cell bodies and central axons can persist for months to years after insult [1–2], while the peripheral fibers and synaptic contacts with hair cells are rapidly and progressively degenerated, presumably due to the sudden and excessive release of glutamate from the presynaptic ribbons of the hair cells during loud noise [3–4]. Damaged synapses between the inner hair cells (IHCs) and SGNs are proposed as a basis for speech-in-noise deficits that may underlie “hidden hearing loss” and are suspected to be a common manifestation of age-related hearing loss [5–6]. Indeed, loss of IHC type 1 afferent fibers and synapses has been shown to occur with age in human subjects [7–8]. This hidden hearing loss can potentially contribute to impaired social, psychological, and cognitive function. Estimates of its prevalence range from 10–12% of adults with otherwise normal hearing [9]. Furthermore, genetic hearing loss can contribute to SGN dysfunction. This includes DFNB1, the most common form of congenital deafness, in which SGN degeneration occurs subsequent to hair cell death, impacting the utility of cochlear implants that rely on healthy SGNs for neurotransmission from the cochlea to the brain [10–11].

The fact that the SGN cell bodies and central axons remain intact for an extended period provides an opportunity to re-establish neuronal function by restoring the type 1 afferent fibers and their synaptic contacts with IHCs. The neurotrophins BDNF and NT-3 activate TrkB and TrkC on SGNs to promote survival, neurite growth, and synapse formation [12]. There is strong evidence supporting neurotrophin therapy for hearing loss, including studies demonstrating that BDNF and NT-3 are critical for establishing synapses during development and maintaining hearing function throughout life [12–13]. Several animal studies have shown that treatment with BDNF or NT-3 has beneficial effects on SGN afferent fibers, synapses, and hearing function after noise or ototoxin-induced hearing loss [14–19].

In this study, we evaluated several monoclonal antibodies and small molecules that are reported to act as TrkB or TrkC receptor agonists for their potential to restore SGN type 1 afferent synapses, and we compared their activity with the native neurotrophins BDNF and NT-3. Each of these agonists has a different activity profile with the potential to influence their therapeutic benefit. In addition, their molecular properties may be important when delivering agents by intratympanic injection for diffusion into the cochlea through the round window membrane. Local delivery of neurotrophic agents to the inner ear is an attractive therapeutic approach that can provide a high local concentration at the target tissues and enables delivery of compounds that have unfavorable ADME or are otherwise inappropriate for systemic administration. While delivery of BDNF or NT-3 to augment the endogenous neurotrophin levels is a logical approach, Trk receptor monoclonal antibody agonists have the potential to be highly selective and have greater biostability than neurotrophins, although their larger molecular size could impede diffusion into the cochlea. Several small molecule TrkB agonists [20–23] are also reported to be highly selective and would be expected to diffuse readily into the inner ear.

For the present studies, seven monoclonal antibodies (M1-M7; referred to as the “M-antibodies”) that bind to Trk receptors and act as agonists were generated. Four antibodies activate TrkB (M3, M4, M5 and M6) and three activate TrkC (M1, M2 and M7). Five of the seven antibodies are humanized (M1, M2, M3, M6 and M7) on an IgG4 backbone, while M4 and M5 are of the mouse IgG1 isotype. We also tested three commercially available small molecules, 7,8-DHF, LM22A4 and LM22B10, which are reported to be Trk agonists. The M-antibodies, neurotrophins BDNF and NT-3, and putative small molecule TrkB agonists were first evaluated for Trk receptor agonist activity in cell lines expressing Trk receptors and then tested in ex vivo assays to evaluate SGN survival, neurite outgrowth and synapse formation. While the small molecules had no effect in our assays, all of the M-antibodies induced Trk receptor activation, and we show that BDNF, NT-3 and the TrkB monoclonal antibody agonist, M3, are capable of driving robust SGN survival, neurite extension, and synapse restoration in an ex vivo model using excitotoxic trauma. These results suggest that BDNF, NT-3 and M3 are promising candidates that should be further investigated for the treatment of hearing loss, including speech-in-noise difficulties.

## Results

### M-antibodies are selective agonists of the TrkB and TrkC receptors

**Fig 1:**
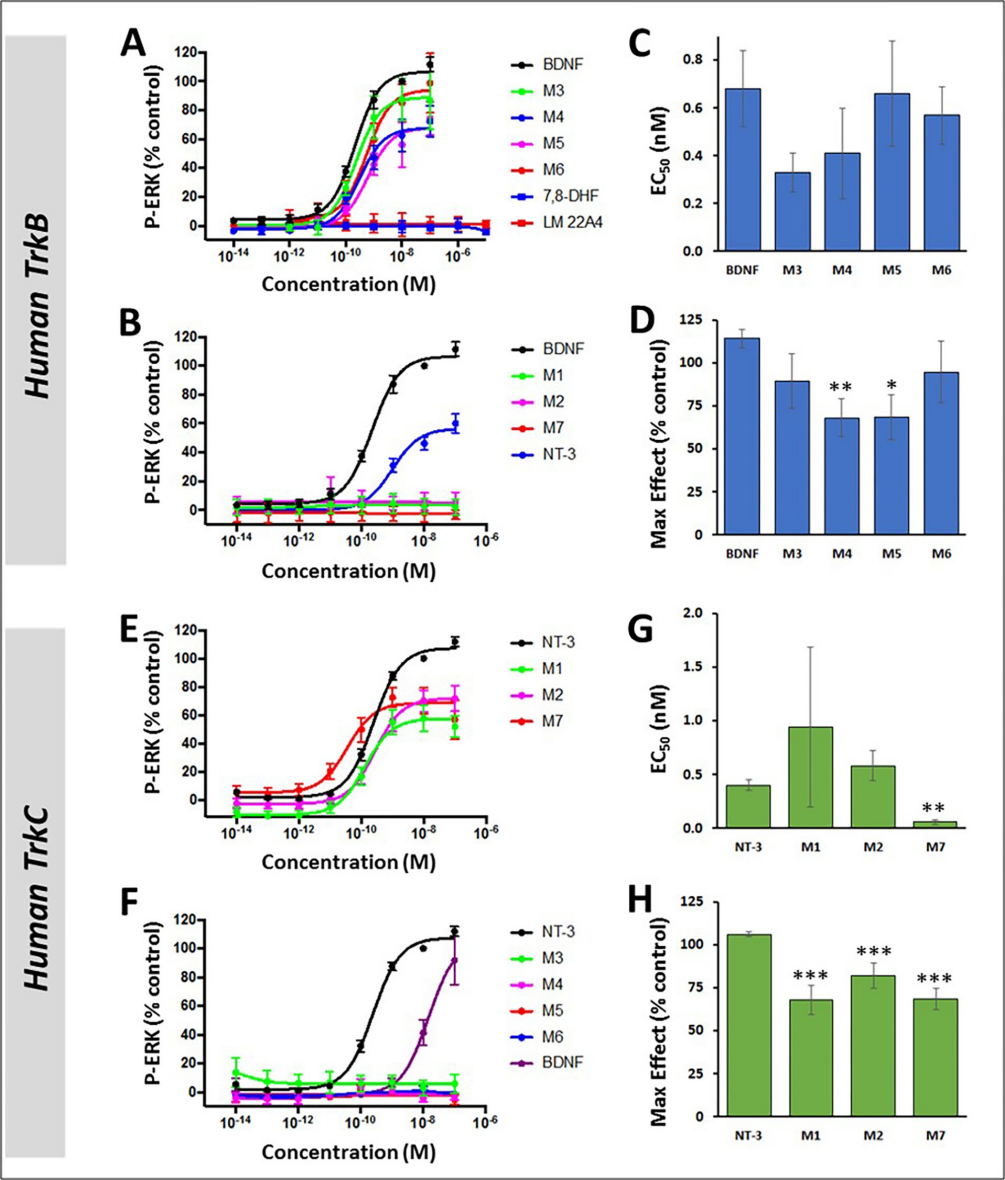
M-antibodies selectively activate Trk receptors as detected by ERK phosphorylation. (**A-B**) In human TrkB-expressing cells, ERK phosphorylation increased dose-dependently in response to M3, M4, M5, M6, BDNF, and NT-3, but not in response to 7,8-DHF, LM22A4, M1, M2, or M7. (**C-D**) The potency (**C**) of M3, M4, M5, and M6 in TrkB-expressing cells was comparable to BDNF, although the maximal effect (**D**) was reduced with M4 and M5. (**E-F**) In human TrkC-expressing cells, phospho-ERK increased dose-dependently in response to M1, M2, M7, NT-3 and BDNF but did not respond to treatment with M3, M4, M5, or M6. (**G-H**) The potency (**G**) of M1, M2, and M7 in TrkC-expressing cells was similar to NT-3, though M7 had a significantly lower EC_50_. Maximal activation (**H**) was significantly reduced with M1, M2, and M7 compared to NT-3. Dose-response curves and max effect bar graphs are normalized to either 10nM BDNF (**A-D**) or 10nM NT-3 (**E-H**). *p<0.01, **p<0.001, ***p<0.0001. Statistical tests were not performed on panels **A**, **B**, **E**, and **F**.

The M-antibodies, neurotrophins, and two small molecule agonists were tested for their ability to selectively activate either TrkB or TrkC using cell lines that stably express exogenous human TrkB or TrkC. To make a quantitative assessment, we used AlphaLISA, an in vitro assay that reports phospho-ERK activity, a common functional readout of Trk activation and mediator of cell survival (Fig 1). The AlphaLISA format provides a quantitative, high capacity alternative to more traditional Western blots, and a comparison of these methods was made in a sub-set of experiments (see S2 Fig). In this optical immunoassay, four of the antibodies (M3, M4, M5, and M6) were potent activators of TrkB, with EC_50_s that were comparable to that of BDNF (ranging 0.33nM-0.68nM, Table 1). The maximum response detected at a saturating dose was similar to BDNF for M3 and M6 but slightly reduced with M4 and M5 (Fig 1A and 1D, and Table 1). NT-3, which is the cognate ligand of TrkC, can also activate TrkA and TrkB with lower potency. In this phospho-ERK assay, NT-3 activated TrkB with an average EC_50_ of 2.05nM and maximal effect of 58.5% compared to BDNF (Fig 1B and Table 1). M1, M2, and M7 did not elicit a response from TrkB at any dose. The small molecules 7,8-DHF and LM22A4, reported TrkB agonists [20, 24, 25], also did not elicit a phospho-ERK response when applied to TrkB-expressing cells, at doses ranging from 0.01pM to 10μM. When applied to TrkC-expressing cells, M1, M2, and M7 provided phospho-ERK responses that were similar to NT-3, with a maximal effect ranging from 68–82% compared to NT-3 (Fig 1E and 1H, and Table 1). The EC_50_ of the M-antibodies in TrkC-expressing cells was similar to NT-3, but M7 was more potent with a 6-fold lower EC_50_ value (0.06nM for M7 vs. 0.40nM for NT-3; Fig 1G and Table 1). At the highest doses, BDNF also elicited a phospho-ERK response in TrkC-expressing cells (Fig 1F and Table 1).

**Table 1:** Effect of M-antibodies on ERK phosphorylation in cell lines expressing human and rat Trk receptors.

		BDNF	NT-3	M1	M2	M3	M4	M5	M6	M7
**TrkB (human) HEK293**	*EC*_*50*_ *nM*	0.68 ± 0.16 (16)	2.05 ± 0.92 (8)	>100 (4)	>100 (4)	0.33 ± 0.08 (4)	0.41 ± 0.19 (5)	0.66 ± 0.22 (5)	0.57 ± 0.12 (4)	>100 (4)
	*Max (%)*	114 ± 5.25	58.5 ± 5.46	NA	NA	89.4 ± 16.0	68.1 ± 11.0	68.7 ± 13.0	94.7 ± 18.0	NA
**TrkB (rat) HEK293**	*EC*_*50*_ *nM*	0.19 ± 0.03 (28)	10.7 ± 3.47 (12)	ND	ND	0.18± 0.05 (11)	0.93 (2)	0.49 (2)	1.77 ± 0.63 (4)	ND
	*Max (%)*	104 ± 2.79	83.0± 17.3	ND	ND	70.4 ± 26.6	61.2	49.5	79.5 ± 2.14	ND
**TrkC (human) NIH3T3**	*EC*_*50*_ *nM*	26.8 ± 10.4 (12)	0.40 ± 0.05 (40)	0.94 ± 0.74 (12)	0.58 ± 0.14 (17)	>100 (4)	>100 (4)	>100 (4)	>100 (5)	0.06 ± 0.02 (11)
	*Max (%)*	104 ± 20.8	106 ± 1.47	67.8 ± 8.64	82.0 ± 7.49	NA	NA	NA	NA	68.2 ± 6.26
**TrkC (rat) NIH3T3**	*EC*_*50*_ *nM*	4.97 (2)	0.21 ± 0.08 (7)	0.65 ± 0.42 (6)	0.67 ± 0.44 (5)	>100 (2)	>100 (2)	>100 (2)	>100 (2)	0.22 ± 0.15 (7)
	*Max (%)*	76.2	103 ± 2.52	56.8 ± 8.16	82.3 ± 5.36	NA	NA	NA	NA	76.9 ± 7.68
		**NGF**	**NT-3**	**BDNF**	**M2**	**M3**	**M4**	**M5**	**M6**	**M7**
**TrkA (human) NIH3T3**	*EC*_*50*_ *nM*	0.97 ± 0.73 (4)	4.35 ± 1.91 (4)	>100 (3)	ND	>100 (3)	ND	ND	ND	ND
	*Max (%)*	106 ± 5.65	73.6 ± 5.08	NA	ND	NA	ND	ND	ND	ND

(**Top**) M-antibodies have similar effects on human and rat Trk receptors as measured by phospho-ERK AlphaLISA. No statistically significant differences were detected when comparing the M-antibodies between the human and corresponding rat cell line. (**Bottom**) A phospho-ERK response was detected in cells expressing TrkA when treated with NGF or NT-3, but not BDNF or M3. ND: Not Done; NA: Not Applicable; Numbers in parentheses indicate the number of times the experiment was performed.

### M-antibodies are cross-reactive between human and rat Trk receptors

While NT-3 and BDNF are 100% conserved across mammals, the Trk receptors differ between species. Human and rat TrkB amino acid sequences are 94% identical, while human and rat TrkC share 97% amino acid identity. In both cases, nearly all differences occur in the extracellular domain, which could potentially impact the ability of the M-antibodies to bind and activate rodent Trk receptors and alter the efficacy of the antibodies in our rat ex vivo cochlear models. The phospho-ERK AlphaLISA assay was used to establish cross-reactivity of the M-antibodies between human and rat species of the Trk receptors, using cell lines that stably express either human or rat Trk receptors. The differences in phospho-ERK activation between species were negligible (Table 1). Furthermore, the TrkB-selective antibody M3 did not activate TrkA in a human TrkA-expressing cell line. The small molecule TrkB agonists 7,8-DHF and LM22A4 were not only inactive as agonists with human TrkB (Fig 1A), but also inactive with the rat TrkB receptor, at concentrations up to 10μM (S1 Fig). In addition, the non-selective TrkB/TrkC small molecule agonist, LM22B10 [23] did not activate rat TrkB (S1 Fig). BDNF and NT-3 elicited a phospho-ERK response from both TrkB and TrkC, but as expected, the effect was weaker in cell lines expressing the non-cognate receptor.

### M-antibodies and neurotrophins increase SGN survival and neurite complexity at nanomolar concentrations

**Fig 2:**
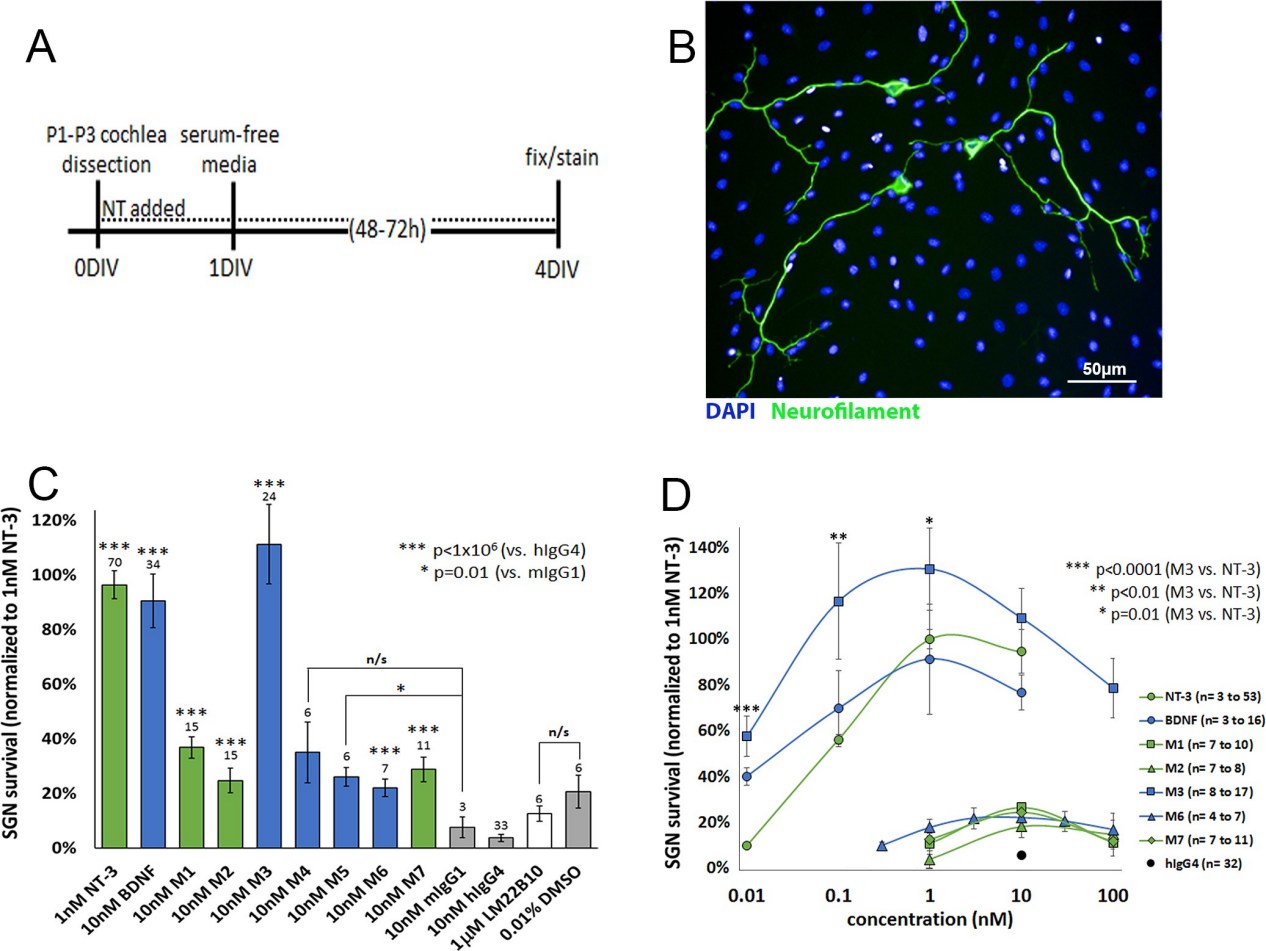
M-antibodies and neurotrophins increase SGN survival. (**A**) Protocol for SGN dissection and culture. NT = neurotrophic agent (NT-3, BDNF, M-antibodies, etc.) (**B**) SGNs were fixed, immunostained for neurofilament (green) and DAPI (blue), and imaged. Surviving neurons were identified as cells with intact soma and at least one neurite. (**C**) The number of surviving SGNs increased when cultured with neurotrophins or M-antibodies, compared to treatment with mouse IgG1 and human IgG4 isotype controls. Survival of SGNs was not improved with LM22B10 compared to a vehicle-only control, 0.01% DMSO. Results are normalized to the number of surviving SGNs in wells treated with 1nM NT-3. Blue bars = TrkB agonist, green bars = TrkC agonist, white bar = non-selective TrkB/TrkC agonist. (**D**) The effect on SGN survival was evaluated across a range of doses, and normalized to 1nM NT-3, as in (**C**). For panel (**C**), N = 3 to 70 wells per treatment condition; for panel (**D**), the numbers of treated wells are provided in the figure key.

Having established that the M-antibodies have strong agonist effects in cell lines expressing Trk receptors and are cross-reactive between rat and human Trk receptors, we next evaluated the effectiveness of these antibodies in eliciting the expected pro-survival responses from SGNs of the cochlea, along with the neurotrophins as well as LM22B10, which is reported to improve survival of hippocampal neurons [23]. SGNs were harvested from neonatal rats, dissociated, seeded into 96-well plates, incubated with neurotrophins, M-antibodies, or LM22B10 for 48–72 hours and then fixed and immunostained. Without growth factors or neurotrophic support, ~96% of SGNs die during the first 48h in culture [26]. As expected, in our experiments, very few surviving neurons were found in wells treated with the isotype control antibodies, mouse IgG1 and human IgG4 (Fig 2C). In contrast, wells treated with BDNF, NT-3, or M-antibodies had significantly more surviving neurons. The greatest effect was observed with M3. The other TrkB-activating antibodies (M4, M5 and M6) also enabled significantly more surviving neurons when compared to their isotype controls but were not as effective as M3 or BDNF. The TrkC-activating antibodies (M1, M2 and M7) significantly improved SGN survival compared to their isotype control but were not as effective as NT-3 (Fig 2C). LM22B10 had no effect on SGN survival at 1μM, a concentration reported to be highly effective at promoting survival of hippocampal neurons [23] (Fig 2C).

We then tested the M-antibodies across a range of doses, using the same SGN survival paradigm (Fig 2D). Both the M-antibodies as well as BDNF and NT-3 displayed a bell-shaped dose-response curve. The TrkC antibodies (M1, M2, and M7) peaked at 10nM. The TrkB antibodies (M3 and M6) showed activity across a broader range of doses, with SGN survival exceeding the human IgG4 isotype control at all doses tested. M3 exceeded BDNF and NT-3 at all of the concentrations tested (0.01nM to 10nM) and had a maximum effect at 1nM, which was 31% greater than the maximum effect of NT-3 (Fig 2D).

**Fig 3:**
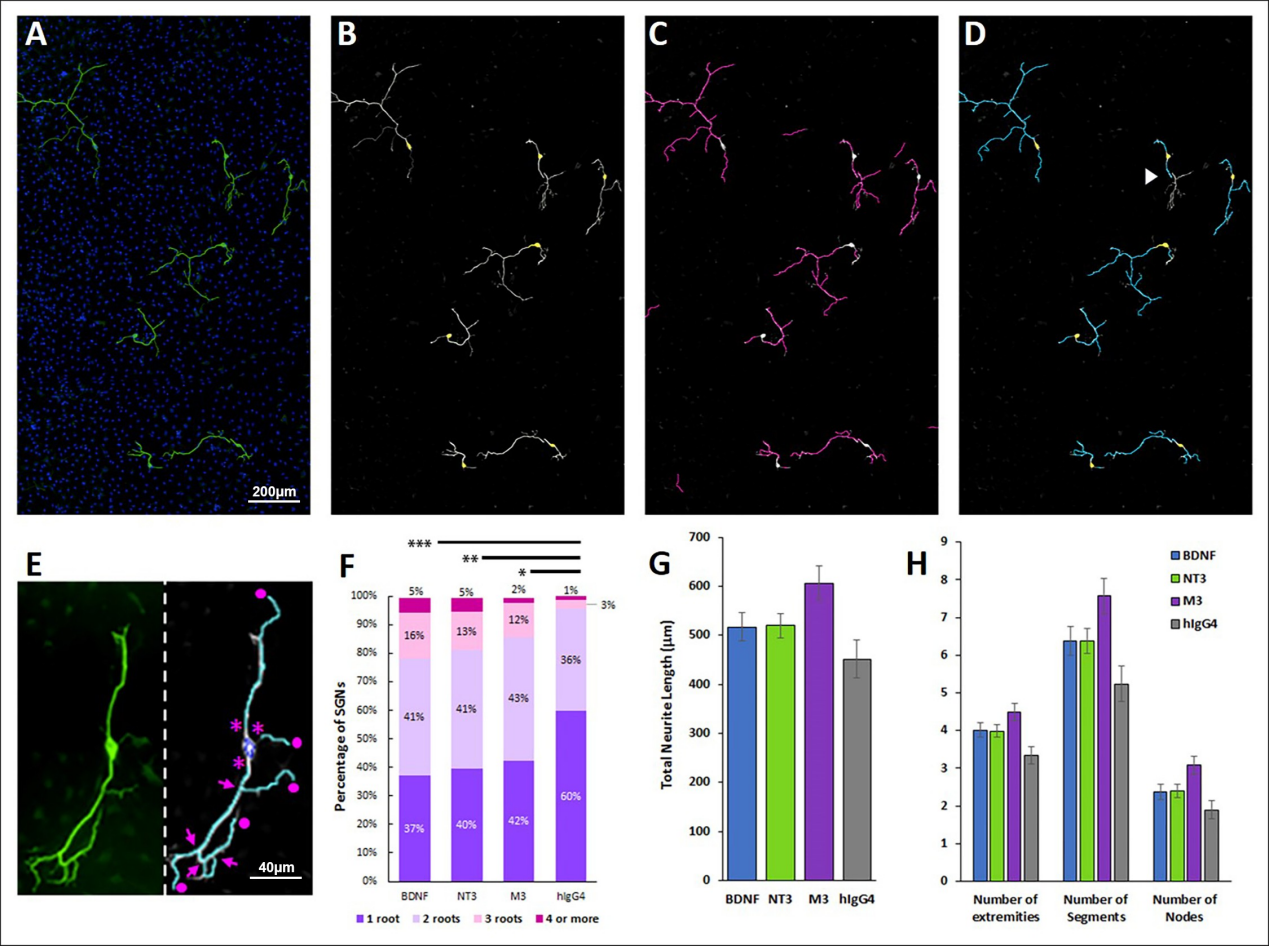
BDNF, NT-3, and M3 increase SGN complexity. (**A-D**) SGNs were immunostained, imaged and batch analyzed using PerkinElmer Harmony 4.6. (**A**) SGNs were immunostained for neurofilament (green) and DAPI (blue) and imaged at 20x. All of the images in a single well were stitched together and analyzed. The analysis detects SGN somas (**B**, highlighted in yellow) and neurites (**C**, magenta). (**D**) Each neurite tree is assigned to a single cell for analysis. Depending on the stitching, occasionally neurites will not meet the criteria due to gaps that exceed a user-defined threshold (**D**, white arrowhead). (**E**) The number of roots (asterisk), extremities (circles), nodes (arrows), segments (discrete sections of neurite between nodes or extremities), and total neurite length were measured for each neuron. (**F**) Treatment with 10nM BDNF, 1nM NT-3, or 1nM M3 increased the number of roots compared to the IgG-treated control. (**G-H**) The total neurite length, as well as number of extremities, segments, and nodes per cell increased with BDNF, NT-3, and M3 treatments compared to hIgG4, but the differences were not statistically significant. *p< 0.05, **p<0.005, ***p<0.001. N = 67 to 834 neurons per treatment condition.

Previous studies have suggested that treatment with neurotrophins impacts not only SGN survival but also SGN morphology [27–28]. We evaluated the effect of BDNF, NT-3, and M3 on dissociated SGN cultures using a neuron detection algorithm (PerkinElmer Harmony 4.6) to provide an unbiased, high-throughput analysis that identifies neurons and measures characteristics, such as number of primary neurites (or roots), bifurcations (nodes), and neurite length. The software stitches together all of the fields in a single well, detects neurofilament-positive cell bodies that have at least one neurite and measures the properties of the affiliated neurite tree. The results are summarized in Fig 3. As expected, BDNF, NT-3, and M3 had a positive effect on neurite outgrowth and complexity compared to the IgG control. 60% of neurons in wells treated with IgG had only one root, whereas a smaller proportion of the BDNF, NT-3, and M3-treated cultures had only one root (37%, 40%, and 42%, respectively), with a corresponding increase in neurons with multiple roots. BDNF, NT-3 and M3 treated cultures also had more extremities, more neurite segments, more nodes, and greater total neurite length, with M3 having the greatest effect, although none of these trends were statistically significant.

### BDNF, NT-3 and M3 promote neurite growth in SGN explants from one-week-old animals

Given that, among the M-antibodies, M3 had the most robust effect on dissociated SGN survival, we then tested it alongside BDNF and NT-3 in a more mature neuronal preparation consisting of explanted spiral ganglion tissues from older, P7-P8 rats [29]. In this model, the spiral ganglion was dissected into ~1mm x 1mm segments that were placed in a 96-well plate. After one day in culture, the media was replaced with serum-free media and treatments were added. Over the course of 72h, neurites extended from the explanted tissue, which was then fixed, immunostained, and imaged.

**Fig 4:**
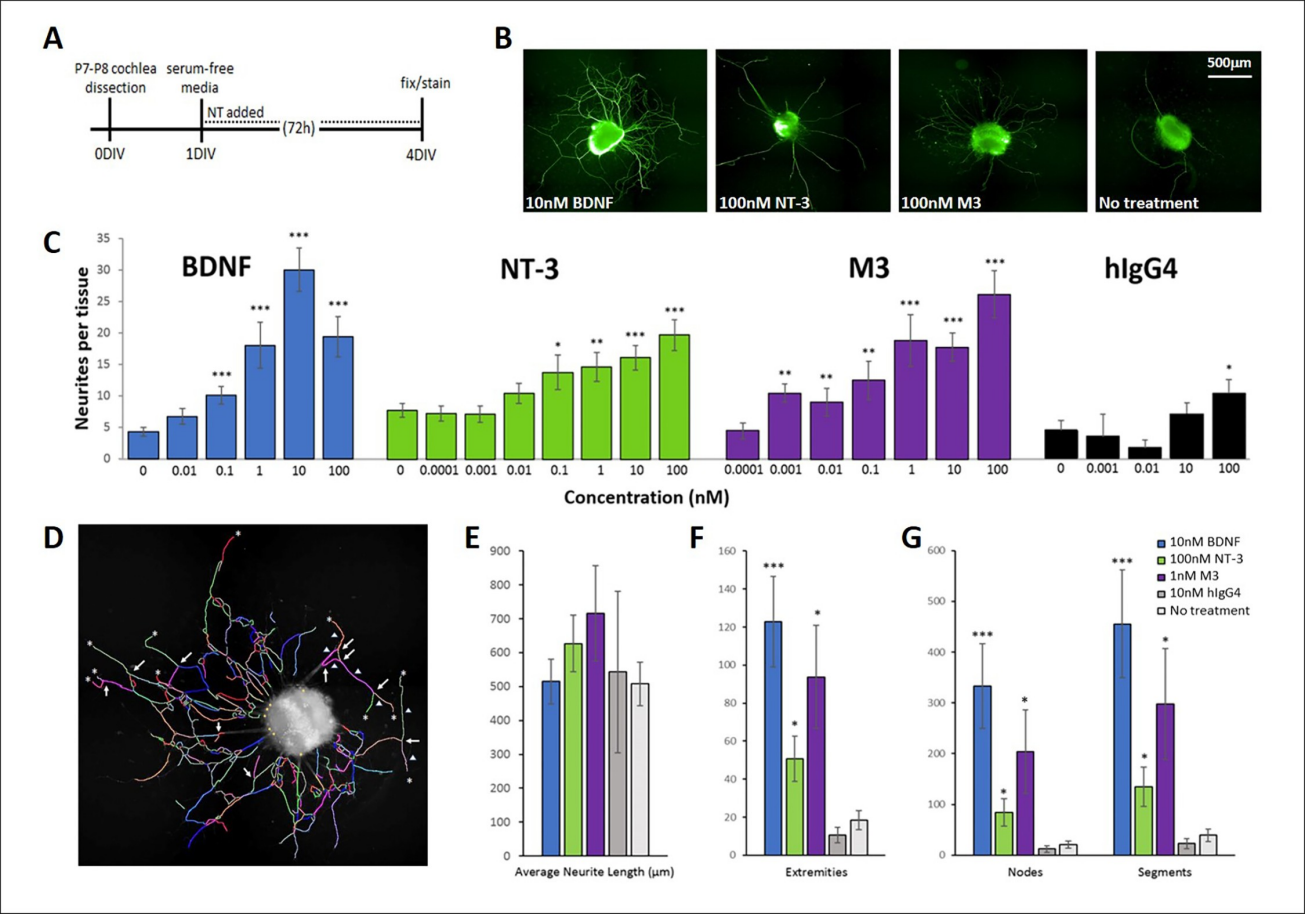
BDNF, NT-3 and M3 promote neurite extension in SGN explants. (**A**) Protocol for explanted SGN dissection and culture. (**B**) Example of SGN explants under different treatment conditions after 48-72h in culture. Neurotrophic treatments increased the number of neurites that extend from the tissue. Tissues were fixed and immunostained for neurofilament (green). (**C**) BDNF, NT-3 and M3 had a dose-dependent effect on the number of neurites per explant. The isotype control, human IgG4, also appeared to promote neurite outgrowth at the highest dose (100nM). (**D**) Explants were imaged and analyzed individually using the Harmony neuron analysis to examine neurite length, nodes (arrows), segments (arrowheads), and the number of extremities (asterisks). (**E-G**) BDNF, NT-3, and M3 had a strong effect on neurite complexity of SGN explants compared to IgG-treated or untreated controls. *p<0.05, **p<0.005, ***p<0.0005. P-values for BDNF, NT-3, and hIgG4 were determined by comparison to untreated SGN explants in the same experiment, and values obtained for M3 were compared to SGN explants treated with 10nM hIgG4 in the same experiment. For BDNF, N = 18 to 50; for NT-3, N = 17 to 50; for M3, N = 19 to 46; for IgG, N = 2 to 37, representing the number of explanted tissues that were combined across multiple independent experiments. For neurite counts, data was combined from nine independent experiments, and for neurite morphology, data was combined from four independent experiments.

The number of neurites per tissue increased dose-dependently with NT-3, BDNF, M3, and to a small extent, with the human IgG4 isotype control (Fig 4C), indicating a possible effect through a non-Trk receptor mechanism. The greatest neurite counts resulted from TrkB stimulation with BDNF or M3, with BDNF having the strongest effect and peaking at 10nM, while M3 was effective across a broad range of doses (1pM-100nM, the highest dose tested). The effect of NT-3 on neurite counts also increased up to the highest dose tested (100nM). The Harmony neuron detection algorithm was used to evaluate morphological characteristics including neurite length, as well as the number of extremities, nodes (branch points) and segments (Fig 4D). Fig 4E–4G shows the peak values, which were obtained after analyzing these properties across all doses (0.01nM to 100nM; see S3–S5 Figs). 10nM BDNF had a maximal effect on neurite number, length, nodes, segments, and extremities. The effect of NT-3 on morphology was dose-dependent and continued to increase even at 100nM. M3 exerted the strongest effects on neurite complexity at 1nM. Of the three agents tested, the TrkB agonists BDNF and M3 showed the greatest effect on neurite count and complexity.

### Neurotrophins and M3 accelerate neuron fiber growth and formation of synapses in intact cochlear explants

**Fig 5:**
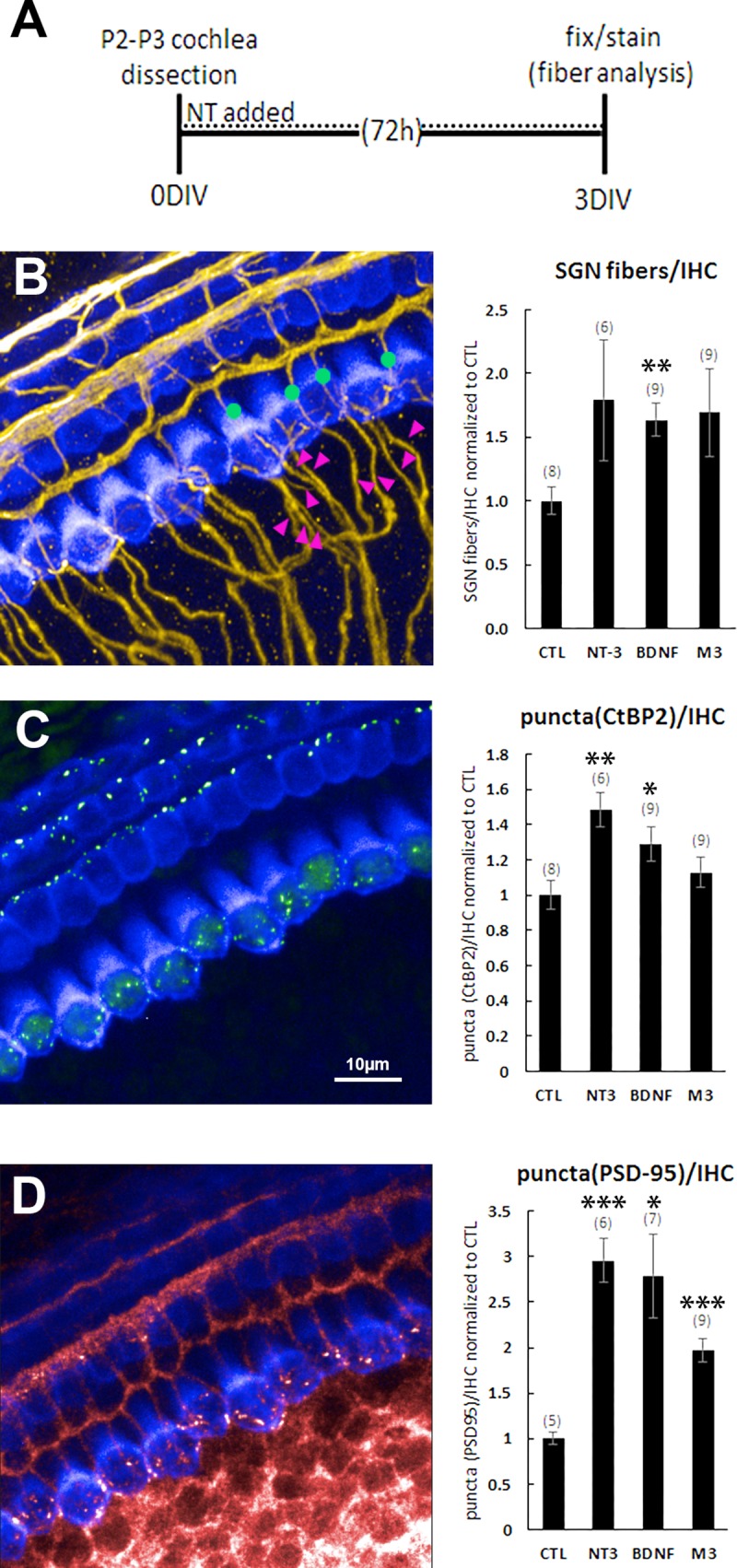
NT-3, BDNF and M3 promote formation of synapses and fiber growth in cochlear explants. (**A**) Protocol for cochlea explantation and culture. (**B**) Explants were immunostained with Myo7A (blue) to identify hair cells and neurofilament (yellow) to label SGNs. The number of type I SGNs was calculated by counting all of the fibers approaching the hair cells and then subtracting those that continue to the outer hair cell layer. Green dots and magenta arrowheads represent a few examples of the fibers that were counted. Neurotrophins and M3 increased the number of type I SGN fibers contacting inner hair cells. (**C**) The pre-synaptic puncta were identified by an antibody that labels CtBP2 (green). Neurotrophins and possibly M3 increased the number of presynaptic puncta. (**D**) The postsynaptic puncta were identified by an antibody that labels PSD-95 (red). Neurotrophins and M3 increased the number of postsynaptic puncta. Bar graphs are normalized to control explants that were maintained in culture media but not treated with neurotrophic agents. The number of explanted tissues is indicated in parentheses and are combined from two (for PSD-95) or three (for SGNs and CtBP2) independent experiments. *p<0.05, **p<0.005, ***p<0.0005 (vs. CTL).

While BDNF, NT-3, and M3 were highly effective at supporting survival and neurite outgrowth in dissociated and explanted SGN cultures, these models do not address whether the treatments will enhance neurite growth toward hair cells and the formation of new synapses. For this, we used a whole cochlear explant culture, in which cochleas were harvested from P2-P3 rats and cultured for several days. This organotypic culture preserves the outer and inner hair cells, the SGNs and their peripheral processes, and the underlying support cells. Upon dissection, cochleas were placed in media containing BDNF (10nM), NT-3 (10nM), or M3 (1nM) and then cultured for 72 hours. Cochlear explants were then fixed and immunostained using Myo7A (to identify hair cells), neurofilament (SGNs), CtBP2 (presynaptic ribbon synapses), and PSD-95 (postsynaptic marker). Neurite number and synapse counts were assessed across a span of 10 or more hair cells from the middle turn of the cochlea. Similar to our results with explanted SGNs, the neurotrophins and M3 promoted neurite growth, measured as the number of SGN fibers contacting inner hair cells (Fig 5A). Furthermore, the number of CtBP2 and PSD-95 puncta were dramatically increased (Fig 5C and 5D), indicating the formation of many new synaptic contacts between the SGN fibers and inner hair cells.

### Neurotrophins and M-antibodies restore SGN fiber density and synaptic puncta in an ex vivo model of noise damage

**Fig 6:**
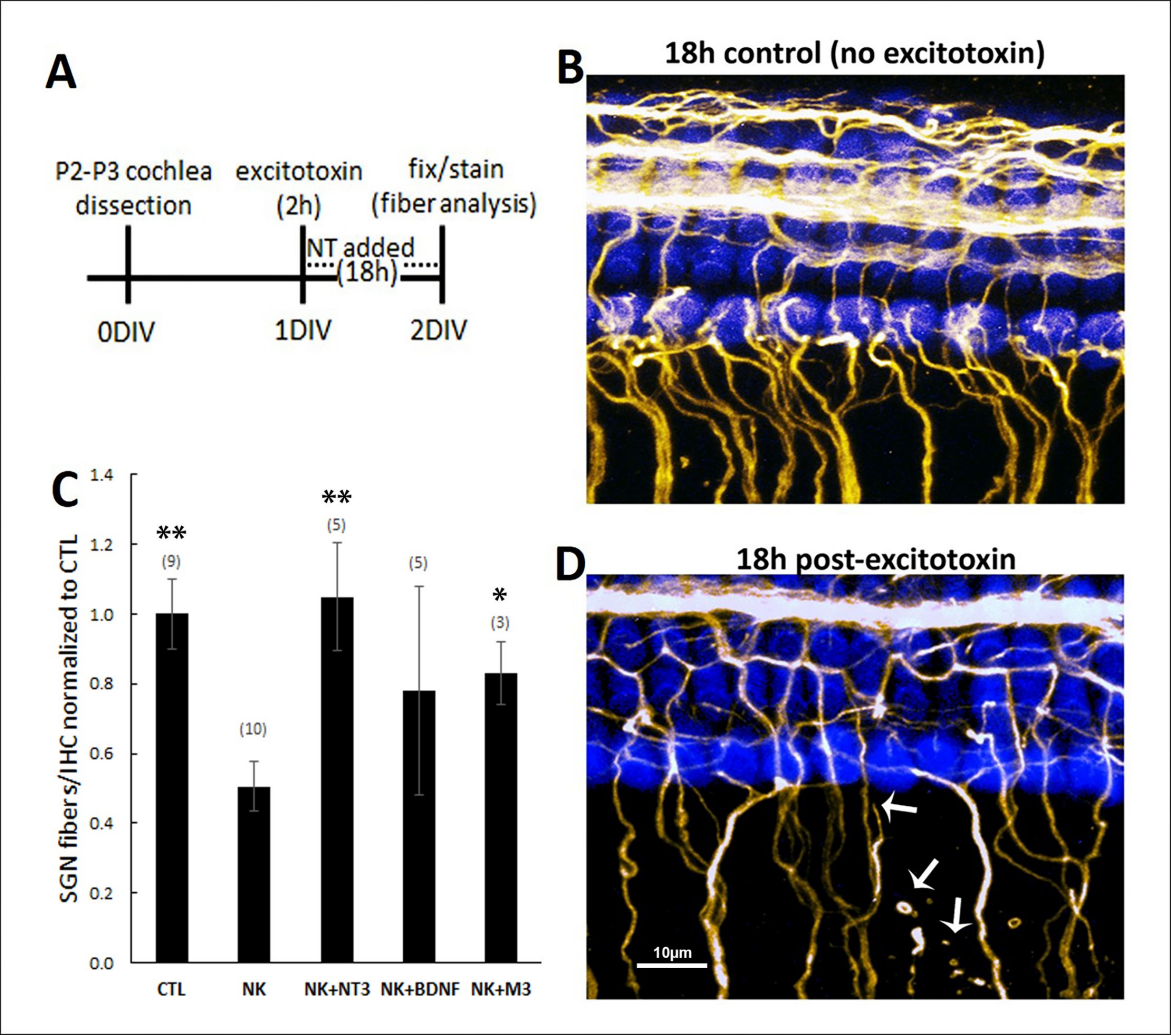
NT-3, BDNF and M3 restore SGN fiber density in organotypic cochlear explants after excitotoxic trauma. (**A**) Protocol for cochlea explantation and culture. (**B,D**) Cochlear explants cultured without excitotoxin (**B**) and with excitotoxin (**D**). Explants were immunostained with Myo7A (blue) to identify hair cells and neurofilament (yellow) to label SGNs. White arrows in (**D**) highlight SGN fibers that are retracting or degenerating. (**C**) Quantification of type I SGNs, calculated as in Fig 5. The number of type I SGNs was reduced with excitotoxin (NK) treatment, but rescued when followed with 1nM NT-3, BDNF, or M3. NK = excitotoxin (NMDA + kainate). The number of explanted tissues is indicated in parentheses and were combined from three independent experiments. *p<0.05, **p<0.005 (vs. NK).

**Fig 7:**
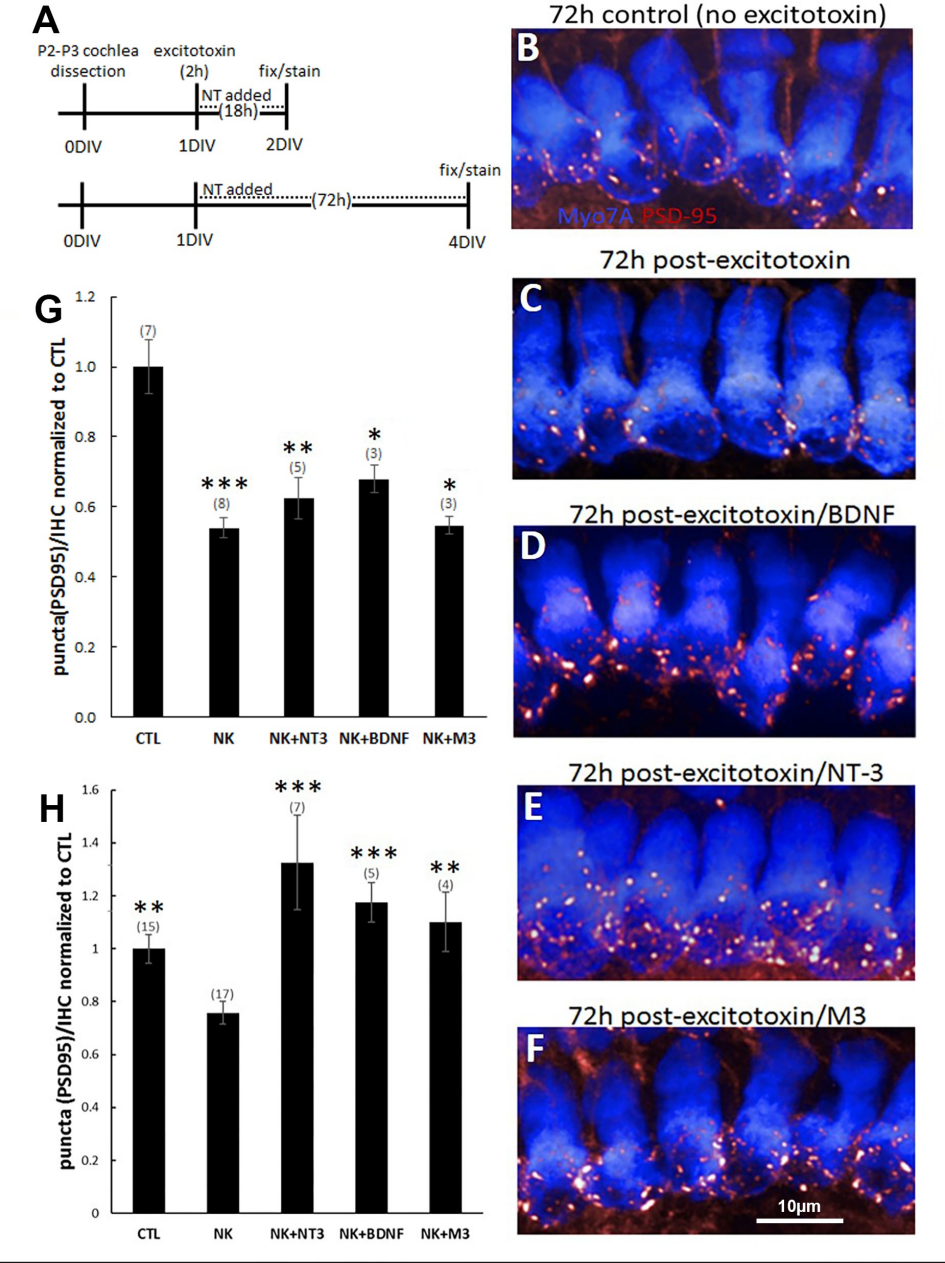
NT-3, BDNF and M3 restore synaptic puncta in organotypic cochlear explants after excitotoxic trauma. (**A**) Protocol for cochlea explantation and culture. Some samples were fixed and immunostained 18h after excitotoxin to evaluate short-term synapse damage. Others were evaluated for restorative effects at 72h post-excitotoxin. (**B**) The number of synapses was measured by counting immunostained PSD-95 puncta (red). Hair cells are labeled with Myo7A (blue). (**C-F**) Example images of puncta counts after excitotoxin: (**C**) without trophic support, (**D**) with BDNF, (**E**) with NT-3, or (**F**) with M3. (**G**) The number of PSD-95 puncta was significantly reduced 18h after excitotoxin (NK) treatment but rescued (**H**) when followed with NT-3 (10nM), BDNF (10nM), or M3 (1nM). The number of explanted tissues is indicated in parentheses and were combined from two (18h timepoint) or five (72h timepoint) independent experiments. *p<0.05, **p<0.005, ***p<0.0005 (vs. CTL in panel G and vs. NK in panel H).

Noise damage to the spiral ganglion neurons is believed to result from the sudden and excessive release of glutamate from hair cells during loud noise, which far exceeds normal synaptic levels. This excitotoxic insult causes the retraction of neuronal processes, the destruction of synapses, and potentially SGN cell death. To test our molecules in an ex vivo model of excitotoxic trauma [30], we cultured organotypic cochlear explants, treated them for two hours with the glutamate receptor agonists NMDA and kainic acid, and then applied neurotrophic agents during the recovery period (Fig 6A). This excitotoxin challenge has been shown to damage SGN fibers and synapses, with effects that can be observed immediately after excitotoxin incubation. In these earlier studies, neurotrophins were applied after excitotoxin for 18-72h and promoted restoration of SGN fibers and synapses [30]. Similarly, in our experiments the type I SGNs, which terminate on the inner hair cells, were reduced in number 18h after excitotoxin application [30], but not if the excitotoxin was followed by treatment with 1nM BDNF, NT-3, or M3 (Fig 6C). We also examined the effect of excitotoxic trauma on the synapses that exist between the inner hair cells and SGNs using an antibody that labels PSD-95 (Fig 7). At 18h post-excitotoxin, the number of PSD-95 puncta was significantly reduced, compared to controls that were not incubated with NMDA and kainic acid, and this degeneration was observed even in the presence of NT-3 (10nM), BDNF (10nM), or M3 (1nM) (Fig 7G). However, by 72h the application of BDNF (10nM), NT-3 (10nM) or M3 (1nM) during the recovery period restored the synaptic puncta to control levels or higher, while a low level of PSD-95 persisted in explants that recovered from excitotoxin in control media containing no treatments (Fig 7H).

## Discussion

Activation of the TrkB and TrkC neurotrophin receptors has been proposed as a promising treatment to reverse cochlear synaptopathy, an injury to the synapses connecting inner hair cells and spiral ganglion neurons that is thought to cause speech-in-noise hearing difficulties in humans. The present study took a systematic approach to evaluate the potential for TrkB and TrkC agonists to activate these receptors, induce downstream signaling, and ultimately produce effects in cochlear tissue that suggest potentially therapeutic benefits. First, the neurotrophins BDNF and NT-3, as well as seven Trk -selective monoclonal antibodies and three proposed TrkB small molecule agonists were tested in cell lines expressing TrkB or TrkC receptors to confirm agonist activity. Second, molecules with confirmed agonist activity were evaluated for their ability to activate native Trk receptors and confer survival to dissociated rat SGNs. Results from these experiments showed that BDNF, NT-3 and the TrkB-specific antibody M3 gave the greatest survival benefit, and these agents were subsequently examined in assays that reflect the desired therapeutic effects of SGN neurite outgrowth and restoration of synapses after injury. BDNF, NT-3 and M3 all induced dose-dependent increases in neurite outgrowth and complexity in one-week-old rat spiral ganglion explants, with BDNF producing the greatest effect. In a rat cochlear explant synaptopathy model, BDNF, NT-3 and M3 restored SGN fibers and synapses with inner hair cells that had been disrupted by excitotoxin treatment. While all of the M-antibodies performed well in cell lines, only the TrkB agonist M3 proved comparable to BDNF and NT-3 in native cochlear tissue. These studies demonstrate that activation of Trk receptors by BDNF, NT-3 and M3 in ex vivo preparations of cochlear tissue promotes the repair of cochlear synaptopathy.

The M-antibodies selectively bound to either TrkB or TrkC with low nanomolar affinity and activated robust phospho-ERK responses that could be easily detected by AlphaLISA. Selectivity was also evident in that antibodies generated by immunization with TrkB (M3, M4, M5, and M6) elicited phospho-ERK responses in cells expressing TrkB but not TrkC, and antibodies generated by immunization with TrkC (M1, M2, M7) evoked phospho-ERK responses in cells expressing TrkC but not TrkB. Further, M3 was not able to elicit a phospho-ERK response in cells expressing TrkA. Overall, these data provided confirmation that the M-antibodies have agonist activity at the appropriate Trk receptors and are highly selective. There are previous reports of agonist antibodies for TrkB [31–36], TrkC [37–39] and TrkA [40–41] that characterized their functional effects in a variety of assays. In general, these antibodies show potent and specific effects on the Trk receptor subtype used in their generation, similar to the data described here. The four TrkB M-antibodies had sub-nanomolar EC_50_ values for hTrkB receptors, equal to that of the cognate ligand BDNF. The TrkC agonist antibodies, M1 and M2, had sub-nanomolar EC_50_ values that were similar to the cognate ligand NT-3, however M7 showed an increased affinity. The basis for a lower maximum response seen with a majority of the M-agonists tested compared with the cognate ligand is not clear. This has been observed with other Trk agonist antibodies [31–35] and sometimes described as a partial agonist response. Neurotrophins exist as dimers that bind to Trk receptors and activate them by promoting and stabilizing Trk homodimers that autophosphorylate though intracellular kinase domains to set in motion intracellular signaling [42–43]. Presumably Trk antibodies that act as agonists do so because they are bivalent and able to promote the formation of a Trk homodimer. They may do this by binding to sites on the Trk extracellular domain that overlap with, or are distinct from, those of the neurotrophins [36]. Therefore, agonist antibodies may stabilize conformations of the Trk homodimer that are different from those formed by the neurotrophins, resulting in sub-maximal effects. Despite this, the maximal response observed may also vary with the signal transduction component measured. Traub et al [36] showed that, compared with BDNF, TrkB agonist antibodies that produced sub-maximal effects on phospho-TrkB and phospho-ERK levels, gave maximal and even supra-maximal effects on phospho-AKT, phospho-CREB and VGF which are downstream signaling elements. In addition, effects observed in cell lines with over-expressed receptors do not necessarily reflect the effects that may occur in native systems. Consequently, we viewed the Trk receptor assay results as a confirmation of agonist activity and selectivity, before testing the molecules in assays using rat SGNs with native Trk receptor and signal cascade expression. In this respect, it was important to also confirm that the M-antibodies had similar Trk agonist activity at rat Trk receptors, so that potential species differences did not confound the results.

The lack of effect of the small molecule TrkB agonists tested conflicts with the original reports of their activity [23, 24, 44] but are in line with more recent papers that have also failed to confirm a direct interaction with TrkB [34, 45, 46]. Others have reported that 7,8-DHF and similar molecules protect SGNs in cochlear explants treated with gentamicin, in the presence of myelin, and in vivo with connexin-26 knockout mice [25, 47, 48]. It is possible that these molecules can produce effects that are independent of TrkB [49]. However, we found a complete lack of activity for LM22B10 in the rat SGN survival assay, indicating that no neurotrophic effect was apparent for cochlear neurons under the present assay conditions where other Trk receptor agonists were clearly effective.

The ability of neurotrophins to support neuronal survival is a cardinal attribute and one that has been used extensively as an assay to determine the effects of Trk receptor agonists in a variety of neuronal populations [50–51]. BDNF and NT-3 support the survival of SGNs in culture through activation of TrkB or TrkC, both of which are expressed in individual SGNs [52–54]. As such, we used dissociated SGNs as a means to evaluate natively expressed Trk receptor responses by assessing their survival, an endpoint that is relevant for the potential clinical use of neurotrophins in inner ear disorders. The M-antibodies gave a significant survival benefit, with all but one peaking at ~20% of the maximum effect induced by NT-3. The exception was the TrkB agonist M3, which gave a survival response equal to or greater than that of NT-3 and BDNF. This effect of M3 could not have been predicted from the results in the cell-based TrkB assay using phospho-ERK as a readout and underlines the value of measuring responses using native cells from the tissue of interest. One possible explanation is that the native molecular environment of the Trk receptors in SGNs may influence the efficacy of the M-antibodies, due to the presence of accessory proteins or co-receptors such as p75. Another possibility is that the M-antibodies are biased agonists that induce differentiated downstream signaling of the Trk receptors. Both BDNF and M3 had very potent survival responses, with significant effects at the lowest dose examined (10pM), and they were effective over a wide concentration range, although the response decreased at the highest concentrations tested (10nM - 100nM). This latter effect has been observed with Trk receptor responses and may be due to Trk receptor desensitization or downregulation [55–57]. Overall, these data suggest that TrkB agonists are potent effectors of SGN survival and may predict a wide therapeutic range for these molecules.

Kondo et al (2013) have shown that rat spiral ganglion explants respond to BDNF and NT-3 by increasing their neurite outgrowth, with the effects of BDNF being more prominent in older explant cultures. Our experiments in P7-8 day explants paralleled these observations since our TrkB agonists, BDNF and M3, gave the most robust response in terms of neurites per explant and increased neurite complexity. This latter aspect was particularly striking for BDNF, which generated 6 to 10-fold more branch points, segments, and extremities than untreated cultures. M3 had a similar but weaker effect (~70% as effective as BDNF) and NT-3 was least effective of the three treatments in this paradigm. The pronounced ability of BDNF and M3 to promote SGN neurite growth could translate to a therapeutic benefit in restoring SGN afferents after they have been damaged by noise or age.

BDNF and NT-3 play prominent roles in establishing type 1 afferent fiber synapses with inner hair cells during development [12–13], and in our experiments using P2-3 cochlear explants, BDNF, M3 and NT-3 all had the ability to increase the number of type 1 afferent fibers and synapses onto inner hair cells. Remarkably, these exogenous neurotrophic agents, which were dispersed throughout the culture media, could augment the normal developmental process and proper targeting, in a manner consistent with endogenous neurotrophin activity when secreted locally from cochlear cells. It is possible that the underlying endogenous neurotrophic tone in these cultures contributed to the fidelity of the exogenous treatments. This targeting by BDNF, M3 and NT-3 also occurred after type 1 afferents and their synapses were disrupted by excitotoxin treatment, a paradigm that mimics the loss of ribbon synapses after noise-trauma or age (4, 30). Taken together, these data suggest that exogenous application of BDNF, NT-3 and M3 may be a means to facilitate reconnection of type 1 cochlear afferent fibers with inner hair cells and provide a therapeutic strategy for addressing synaptopathy that has been associated with hidden hearing loss [58].

BDNF and NT-3 have previously undergone clinical evaluation as treatments for neurological disorders [59–62]. No marked therapeutic benefit was observed in these studies, although the neurotrophins had a very favorable safety profile, even when delivered at high doses directly to the CNS. In some studies, the neurotrophins were administered systemically, so their potential therapeutic effects in the brain were likely compromised by the rapid degradation of these proteins in the blood and by an inability to cross the blood-brain barrier [63]. Even when administered locally to the brain or spinal cord, penetration through CNS tissue to the vulnerable cells was also likely an issue. A more favorable outlook exists for local delivery of therapeutics to the cochlea, since the round window membrane has been shown to provide access for both small and large molecules when applied to its surface from the middle ear [64]. Once across the round window membrane and into the perilymph, molecules have direct access to their target cells. In the case of the neurotrophins, these target cells are the SGN cell bodies and cochlear afferents that express TrkB or TrkC on their surface. Indeed, several studies have shown beneficial effects of BDNF or NT-3 applied to the round window membrane in animal models where SGN function has been compromised by ototoxins or noise [14, 18, 19] In our previous studies, BDNF was administered to the round window membrane by intra-tympanic injection of a thermosensitive sustained-exposure formulation, in a rat model of noise-induced hearing loss and found to provide significant structural and functional amelioration of cochlear synaptopathy [65]. Taken together, these in vivo findings from multiple investigators indicate that that effects of neurotrophins observed in the early post-natal explant models appear to translate into the adult condition. The known safety of BDNF and NT-3 from human studies, combined with the potential for effective local delivery to the inner ear, presents an attractive approach that should be evaluated clinically in hearing disorders, such as hidden hearing loss, where a neurotrophic mechanism may be beneficial.

In conclusion, after evaluating several classes of neurotrophic agents for TrkB and TrkC agonist activity in cell based assays and for their physiological effects in excised rat cochlear tissue, the neurotrophins BDNF, NT-3 and a monoclonal antibody TrkB agonist, M3, emerged as having robust effects on SGN survival, SGN neurite outgrowth and complexity, and the ability to reconnect cochlear afferent fibers with inner hair cells in an ex vivo model that mimics noise or age-related cochlear synaptopathy. Our results pave the way for additional development of BDNF and M3, including an assessment of biostability, bioavailability and efficacy in animal models with damaged SGN type 1 afferents and synapses, to further establish their potential to treat hidden hearing loss and other clinical paradigms that would benefit from restoring these synapses.

## Materials and methods

### Monoclonal antibodies

Trk receptor agonist antibodies M1-M7 (Patent application #15222764 [66]) were generated by cloning the variable region sequences into vectors containing the human IgG4κ backbone containing the hinge stabilizing mutation S108P in the heavy chain then transiently transfected into HEK 293 cells. The antibodies were purified from the supernatants using protein A affinity chromatography, and characterized by SDS-PAGE and size-exclusion chromatography.

### AlphaLISA

#### Cells and incubation with test antibodies

Cell lines stably expressing human TrkB or TrkC (in an HEK293 or 3T3 cell background, respectively) were maintained in culture with Dulbecco’s modified Eagle’s medium (DMEM; Thermo Fisher catalog# 10564–011) with 10% fetal bovine serum and 1% antibiotic-antimycotic (Thermo Fisher catalog# 15240062). 48 hours prior to assay, cells were transferred to a 96-well plate (8000 cells/well for HEK293 cells; 2500 cells/well for 3T3 cells). On the day of the assay, cells were serum-starved by replacing the culture medium with DMEM(100μl/well) and incubated for 1–4 hours at 37°C. Cells were then incubated (with BDNF, NT-3 M-antibodies, and small molecule agonists for 20 min in phosphate-buffered saline (PBS) at room temperature. After aspiration of the incubation media, cells were lysed by the addition of lysis buffer (1x AlphaSure Lysis Buffer Ultra, Perkin Elmer; 100μl/well for 3T3 cells and 50μl/well for HEK293 cells). The lysates were frozen at -80°C until at least the following day.

#### Determination of phospho-ERK (p-ERK) using AlphaLISA

10μl of the cell lysates were placed in a 384-well plate. 10μl of antibody reagents were added to each well followed by 10μl of the acceptor/donor bead mix according to the kit instructions (Perkin Elmer kit: ALSU-PERK-A10K). Incubation was continued overnight/16h at room temperature in the dark and the 384-well plates were read at 680/520-620 excitation/emission using an Enspire (Perkin Elmer) plate reader. RLU (relative light units) values obtained are a quantitative representation of p-ERK in the cells. After subtraction of background RLU values from untreated cells, values for test agents were expressed relative to that for 10nM BDNF or 10nM NT-3 (100%) for TrkB or TrkC, respectively, and dose-response curves were generated in GraphPad Prism. EC_50_ and maximum effect values were calculated for individual dose-response curves using a curve fitting program in GraphPad Prism.

### Dissociated SGNs

Postnatal Sprague Dawley rats (P2-4) of both sexes were anesthetized with isoflurane and decapitated. Temporal bones were removed and transferred to a cell culture dish containing dissection medium consisting of HBSS (Thermo fisher Scientific; catalog# 14175079), 1M HEPES (Sigma; catalog# 83264) and 0.35% D-glucose (Fisher Scientific; catalog# D16 500). Under microscopic visualization, the cochlear capsule was carefully removed from the temporal bone using forceps and transferred to a new cell culture dish containing dissection medium. The cochlea was then removed from the cochlear capsule using fine forceps. The stria vascularis, Reissner’s membrane and the organ of Corti were separated from the cochlear tissue, and the spiral ganglion neurons were subsequently detached from the modiolus. This strand, containing spiral ganglion neurons, was transferred to a 1.5mL microcentrifuge tube containing 0.9mL of dissection medium. Once ~12 of these strands (representing 6 animals) were collected, enzymatic and mechanical dissociation proceeded as described below.

0.1ml of Trypsin (Gibco catalog# 15090–046) was added to the spiral ganglion neurons in HBSS dissection medium and incubated at room temperature for 10 minutes. The cells were then briefly centrifuged, the supernatant discarded, and the cells were washed twice with culture medium (containing DMEM (Life Technologies, catalog# 10564029), N2 supplement (Thermo Fisher catalog# 17502001) and 10% fetal bovine serum (Thermo Fisher catalog# A31604-02)). The cells were resuspended in 0.5ml of culture medium and mechanically dissociated with a glass pipette, for 4–6 triturations. After 4–6 triturations, the cells were briefly centrifuged, and the supernatant applied to a 40μm cell strainer (Corning Falcon catalog# 352340). This was repeated until the tissue was fully dissociated with no visible cell clusters remaining. Surviving cells were counted using the Life Technologies Countess II (AMQAX 1000) using trypan blue, and then seeded into a 96-well plate (Corning, catalog# 62405–439) at a density of 1.4 x 104 cells per well.

At the end of the experiment, cells were fixed in cold 4% paraformaldehyde for 20 minutes, then washed twice in PBS containing 0.5% triton (PBS-T). Cells were then incubated 1-2h at room temperature in primary antibody (Anti-200 kD chicken-Neurofilament Heavy antibody; Abcam catalog# ab134459) in PBS-T containing 10% goat serum. After washing three times in PBS, the cells were then incubated for 1h at room temperature with secondary antibody (Goat Anti-Chicken IgY H&L (Alexa Fluor® 488) preadsorbed; Abcam catalog# ab150169). Cells were then washed twice, treated with DAPI nuclear stain for 5–10 minutes, and then washed two more times with PBS before imaging. Numbers of SGNs (identified by neurofilament staining) surviving in each well were counted. Harmony 4.6 was used for neurite analysis and estimation of neurite properties.

### Explanted SGNs

Temporal bones were obtained from P7-8 Sprague-Dawley rat pups and further dissected in HBSS. To harvest the SGNs, the cochlear capsule was cut open, the modiolus was excised and the organ of Corti was peeled away. The modiolus was then longitudinally split to expose the spiral ganglion region. Thin bony tissues covering the spiral ganglion region were carefully removed and ganglion tissue was cut into ~1mm sections such that 6–8 pieces were obtained from one ear. Each tissue section was cultured in a single well of a human fibronectin coated 96-well plate (Corning catalog# 62405) containing DMEM (Thermo fisher catalog# 10564029), 10% FBS and N2 supplement, such that each group receives tissues from apex, mid and basal region of the spiral ganglion neuron from multiple ears. After culturing the tissues at 37°C and 5% CO2 for approximately 24h, the media was replaced with serum free media and neurotrophic factors were added in the concentrations mentioned. Tissues were then cultured for an additional 72h for neurite outgrowth and then fixed with 4% PFA and immunostained using neurofilament (Abcam catalog# ab134459) and DAPI. Post immunostaining, the plate was imaged using Perkin Elmer-Operetta at 10X magnification. The number of primary neurites outgrowing from each explant was detected by neurofilament staining and were counted manually. Harmony 4.6 phenoLOGIC was used for neurite analysis and estimation of neurite properties.

### Cochlear explants

Temporal bones were obtained from P2-3 Sprague-Dawley rat pups and further dissected in HBSS. The cochlear capsule was cut open, the modiolus was excised, and the remaining tissue was placed on a porous cell culture insert (Millipore, catalog # PICMORG50) in 1mL of media. The media consisted of DMEM (Gibco, catalog #10564), 10% FBS, N2 supplement, penicillin, amphotericin B (Gibco, catalog #15290018). Explants were established in culture for 18-24h prior to the experiments. For excitotoxic insult, explants were exposed to 0.5 mM NMDA (Tocris, catalog# 0114) + 0.5 mM kainate (Tocris, catalog#0222) for 2h, followed by the addition of neurotrophins/antibodies after the excitotoxin was removed (NT-3, BDNF = 10 nM; M3 = 1 nM). Explants were then cultured for 18 or 72h prior to fixation (4% PFA) and immunostaining for Myosin7a (Proteus, catalog #25–6790), CtBP2 (BD Biosciences, catalog# 612044), PSD-95 (Millipore, catalog # MABN68) and neurofilament (Abcam, catalog# ab134459).

### Statistics

An unpaired Student’s t-test was used to compare the means of each experimental condition to its appropriate control, as described in the figures. The two-tailed p-values are reported in the figures and figure legends. If the p-value was greater than 0.05, then the differences were not considered statistically significant. Error bars represent the standard error of the mean (SEM). All data are presented as the means of biological replicates, with the exception of Figs 2 and 3. In Fig 2, after counting surviving neurons, each well was normalized to the neuron count from wells treated with 1nM NT-3 within the same experiment, and then the normalized counts from all wells receiving a particular treatment were averaged across multiple experiments. In Fig 3, individual neurons were analyzed, and their properties were averaged across multiple experiments.

## Supporting information

S1 Fig The reported small molecule agonists 7,8-DHF, LM22A4 and LM22B10 did not activate phospho-ERK responses from cells expressing rat TrkB in the AlphaLISA assay. (TIF)

S2 Fig BDNF stimulates p-ERK in HEK293 cells expressing rat TrkB in a dose-dependent manner as detected by automated Western blot. Cells were treated exactly as described for the AlphaLISA assays, which included incubation for 20 min with a range of BDNF concentrations or IgG (negative control) and lysis. The lysate was then used for automated Western blot analysis using the Wes system. (**A**) Virtual Western blot obtained on the automated Wes system using a p-ERK antibody on lysates from BDNF or IgG-treated HEK293 cells. Note the faint bands in the untreated and IgG (negative control) lanes and the increase in band intensity with increasing concentrations of BDNF. (**B**) Virtual Western blot of total ERK using the same lysates as (A). (**C**) Quantification of p-ERK as visualized in (A). Note the low RLU values in the untreated and IgG samples. (**D**) The data for p-ERK and total ERK in the samples, expressed as percent ERK phosphorylation. Note that the percent of phosphorylated ERK reaches a maximum of approximately 60%, and the IgG negative control has a value of approximately 5%.
(TIF)

S3 Fig Average neurite length, number of nodes, number of extremities and number of segments were determined for SGN explants that were treated with NT-3 at concentrations up to 100nM. *p<0.05, **p<0.005, ***p<0.0005 (vs. no NT-3).
(TIF)

S4 Fig Average neurite length, number of nodes, number of extremities and number of segments were determined for SGN explants that were treated with BDNF at concentrations up to 100nM. *p<0.05, **p<0.005, ***p<0.0005 (vs. no BDNF).
(TIF)

S5 Fig Average neurite length, number of nodes, number of extremities and number of segments were determined for SGN explants that were treated with M3 at concentrations up to 100nM. *p<0.05, **p<0.005, ***p<0.0005 (vs. hIgG4).
(TIF)
