# Supplementary figures and images for: BDNF, NT-3 and Trk receptor agonist monoclonal antibodies promote neuron survival, neurite extension, and synapse restoration in rat cochlea ex vivo models relevant for hidden hearing loss
Source: PLoS One. 2019 Oct 31;14(10):e0224022. doi: 10.1371/journal.pone.0224022 (PMC6822712; doi:10.1371/journal.pone.0224022)

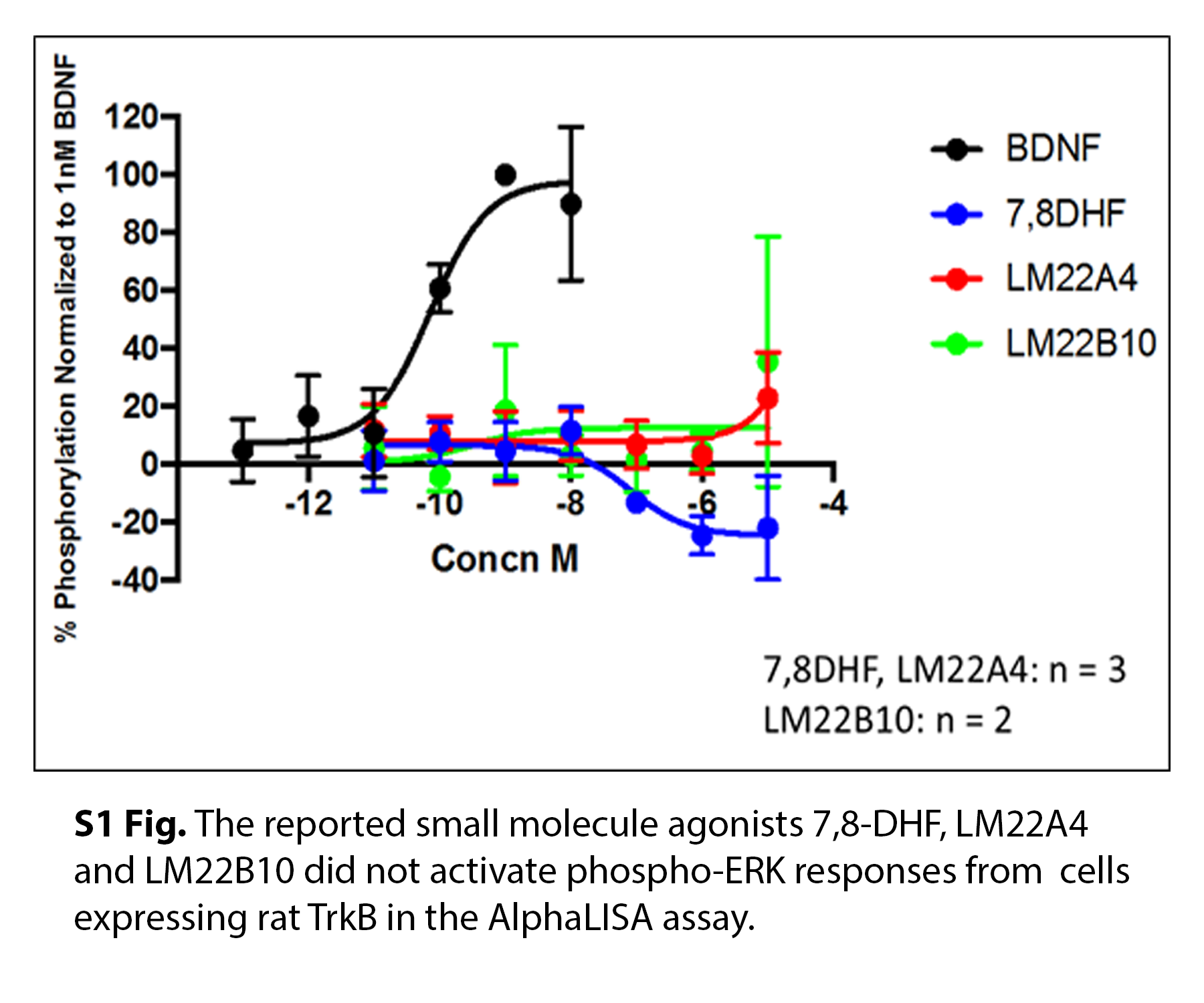

Supplement: S1 Fig — (TIF) [file pone.0224022.s001.tif]

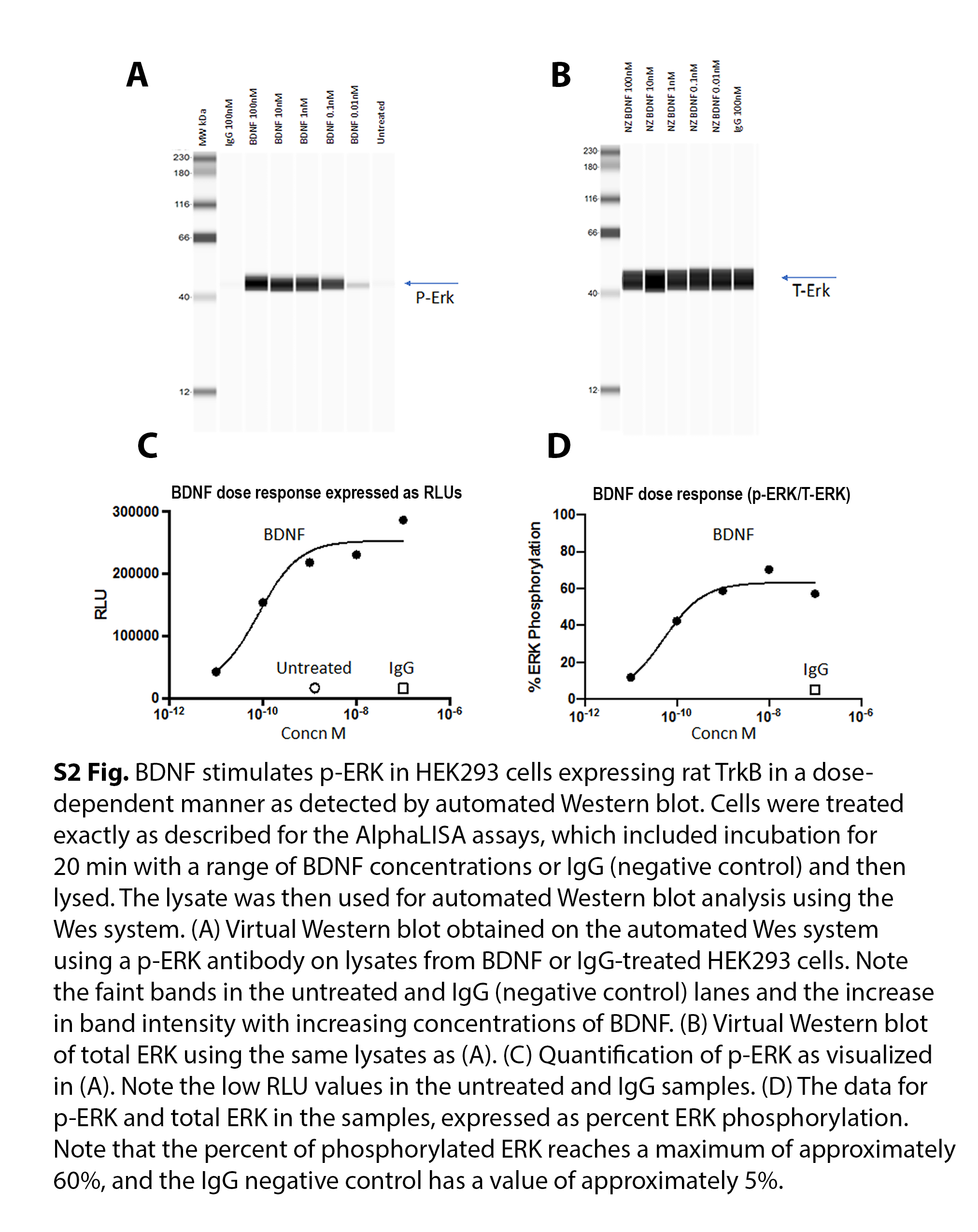

Supplement: S2 Fig — Cells were treated exactly as described for the AlphaLISA assays, which included incubation for 20 min with a range of BDNF concentrations or IgG (negative control) and lysis. The lysate was then used for automated Western blot analysis using the Wes system. (A) Virtual Western blot obtained on the automated Wes system using a p-ERK antibody on lysates from BDNF or IgG-treated HEK293 cells. Note the faint bands in the untreated and IgG (negative control) lanes and the increase in band intensity with increasing concentrations of BDNF. (B) Virtual Western blot of total ERK using the same lysates as (A). (C) Quantification of p-ERK as visualized in (A). Note the low RLU values in the untreated and IgG samples. (D) The data for p-ERK and total ERK in the samples, expressed as percent ERK phosphorylation. Note that the percent of phosphorylated ERK reaches a maximum of approximately 60%, and the IgG negative control has a value of approximately 5%. (TIF) [file pone.0224022.s002.tif]

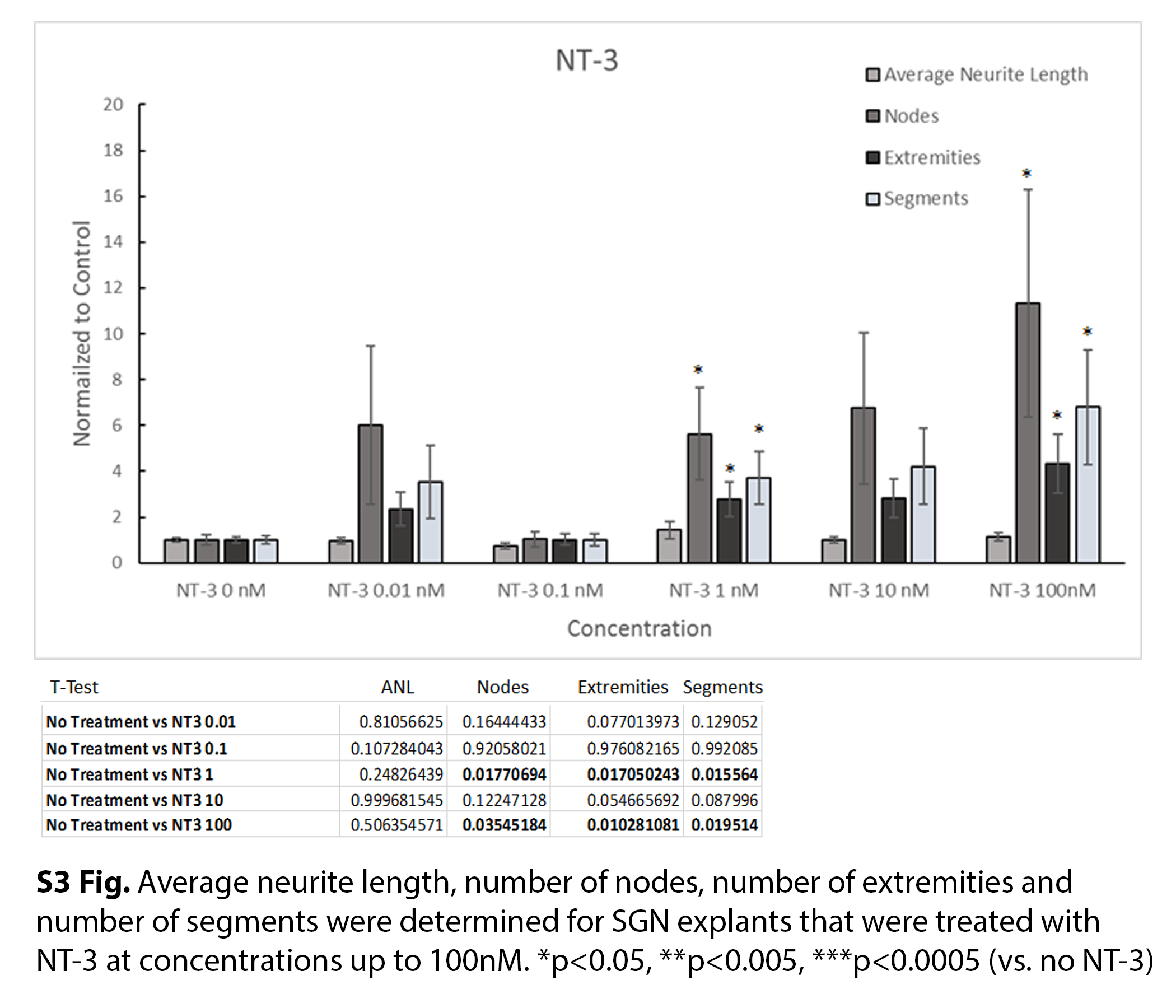

Supplement: S3 Fig — *p<0.05, **p<0.005, ***p<0.0005 (vs. no NT-3). (TIF) [file pone.0224022.s003.tif]

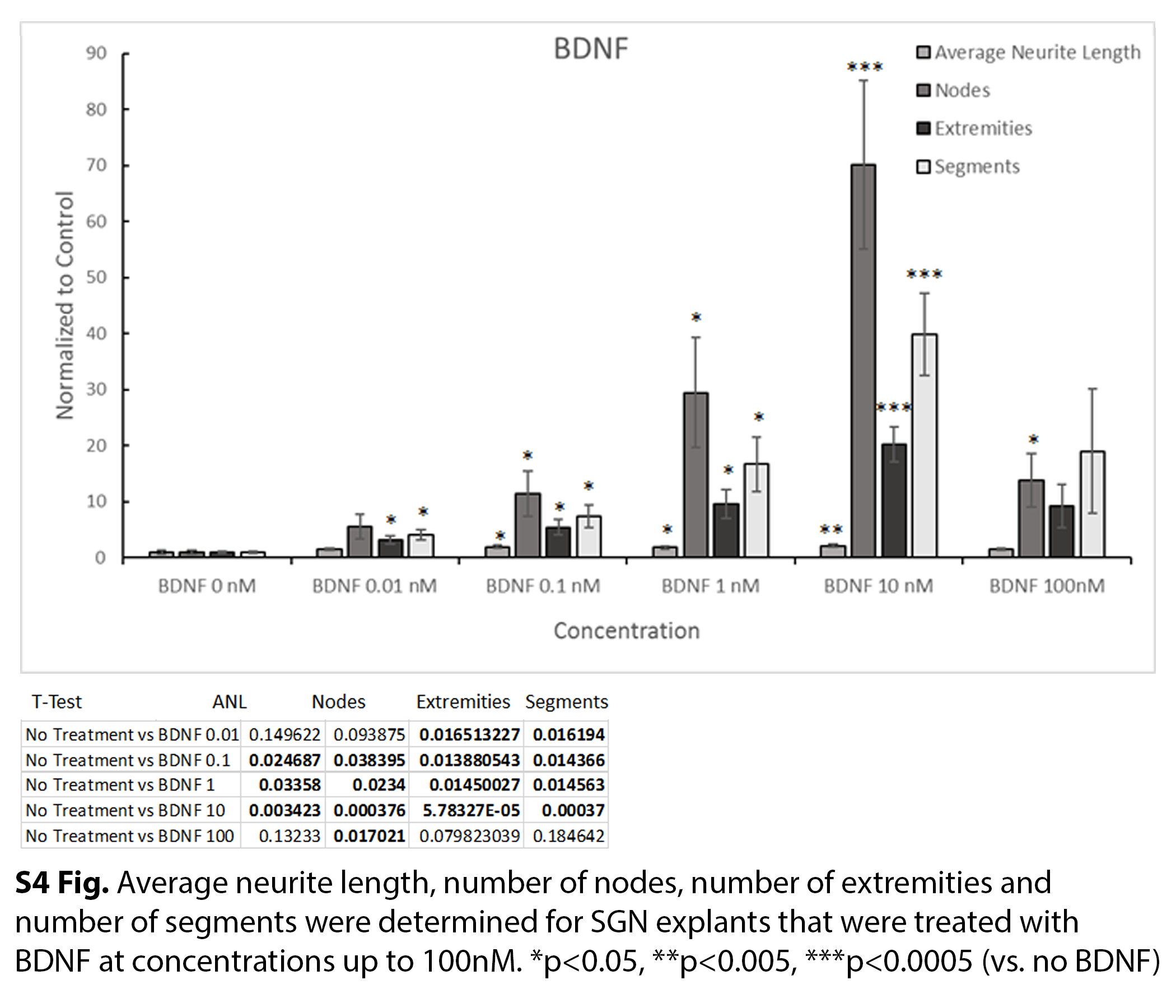

Supplement: S4 Fig — *p<0.05, **p<0.005, ***p<0.0005 (vs. no BDNF). (TIF) [file pone.0224022.s004.tif]

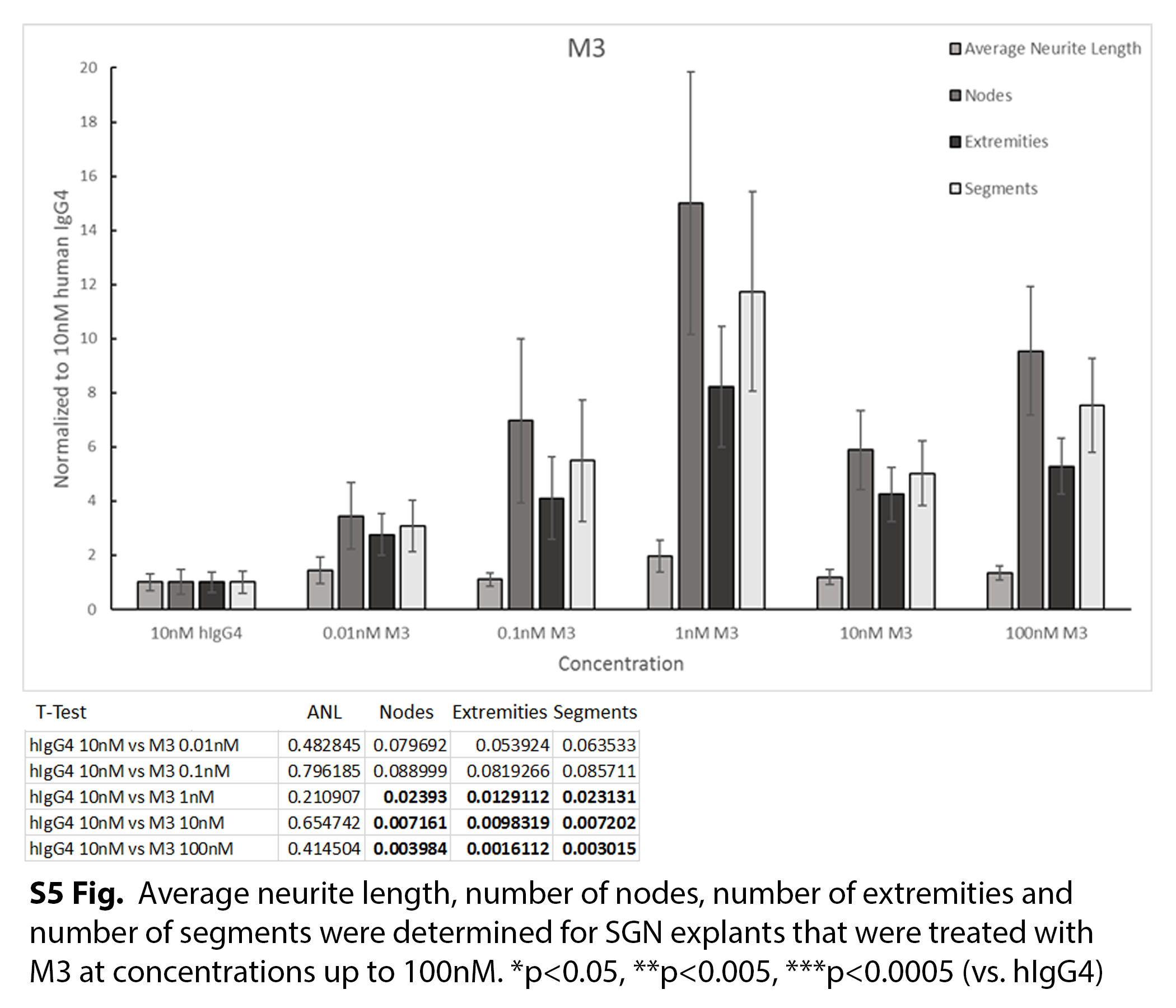

Supplement: S5 Fig — *p<0.05, **p<0.005, ***p<0.0005 (vs. hIgG4). (TIF) [file pone.0224022.s005.tif]
